# A Critical Examination of Senior Housing: Promoting Equity Through Ensuring Safe Spaces

**DOI:** 10.1093/geroni/igab046.1539

**Published:** 2021-12-17

**Authors:** Tam Perry, Zach Kilgore, Michael Appel, Michele Waktins, Claudia Sanford, Dennis Archambault

**Affiliations:** 1 Wayne State University, Detroit, Michigan, United States; 2 CSI Support & Development, Warren, Michigan, United States; 3 Develop Detroit, Detroit, Michigan, United States; 4 Volunteers of America-Michigan, Southfield, Michigan, United States; 5 United Community Housing Coalition, Detroit, Michigan, United States; 6 Authority Health, Detroit, Michigan, United States

## Abstract

As affordable senior housing communities aimed to address the health and well-being concerns of residents in the COVID-19 pandemic, special attention to safety during renovation had to be addressed. This paper offers case studies from members of a city-wide advocacy group, Senior Housing Preservation-Detroit. Eighty one percent of covid deaths in the City of Detroit are those 60 and above; 81.2% of deaths have been among African Americans (Detroit Health Department, 2021). With the grief and challenge in a city hit early on in the 2020 pandemic, these case studies will highlight how Covid-19 affected planned projects in senior buildings, how stakeholders such as developers, staff and residents responded and key considerations for future emergencies affecting senior housing communities. This paper offers critical perspectives applicable to many urban landscapes in order to raise awareness to policy makers, and practitioners.

